# Determining the target protein localization in 3D using the combination of FIB-SEM and APEX2

**DOI:** 10.1007/s41048-017-0043-x

**Published:** 2017-11-04

**Authors:** Yang Shi, Li Wang, Jianguo Zhang, Yujia Zhai, Fei Sun

**Affiliations:** 1 National Key Laboratory of Biomacromolecules, CAS Center for Excellence in Biomacromolecules, Institute of Biophysics, Chinese Academy of Sciences, Beijing, 100101 China; 2 Center for Biological Imaging, Institute of Biophysics, Chinese Academy of Sciences, Beijing, 100101 China; 3 University of Chinese Academy of Sciences, Beijing, 100049 China; 4 Sino-Danish Center for Education and Research, Beijing, 100190 China

**Keywords:** Enhanced ascorbate peroxidase, Focus ion beam scanning electron microscopy, Mitochondrial dynamics, Protein location, Three-dimensional space

## Abstract

Determining the cellular localization of proteins of interest at nanometer resolution is necessary for elucidating their functions. Besides super-resolution fluorescence microscopy, conventional electron microscopy (EM) combined with immunolabeling or clonable EM tags provides a unique approach to correlate protein localization information and cellular ultrastructural information. However, there are still rare cases of such correlation in three-dimensional (3D) spaces. Here, we developed an approach by combining the focus ion beam scanning electron microscopy (FIB-SEM) and a promising clonable EM tag APEX2 (an enhanced ascorbate peroxidase 2) to determine the target protein localization within 3D cellular ultrastructural context. We further utilized this approach to study the 3D localization of mitochondrial dynamics-related proteins (MiD49/51, Mff, Fis1, and Mfn2) in the cells where the target proteins were overexpressed. We found that all the target proteins were located at the surface of the mitochondrial outer membrane accompanying with mitochondrial clusters. Mid49/51, Mff, and hFis1 spread widely around the mitochondrial surface while Mfn2 only exists at the contact sites.

**Electronic supplementary material:**

The online version of this article (doi:10.1007/s41048-017-0043-x) contains supplementary material, which is available to authorized users.

## Introduction

Protein localization correlates with its particular function in cells or tissues. Mapping protein localization information onto their cellular ultrastructural context is of great importance for cell biology study and can be achieved via electron microscopy (EM). Generally, there are two ways to localize a target protein in EM: immune-localization and clonable tags localization. The EM contrast of immune-localization comes from the antibody-conjugated gold particles (De Mey *et al.* 1981) or quantum dots (Giepmans *et al.* 2005). This approach is significantly limited due to the limited efficiency of immunolabeling, the spatial hindrance of large antibodies, and the fact that the well-preserved ultrastructure and the antigen immuno-activity are always mutually exclusive.

Clonable tags localization has been achieved via metallothionein, a small cysteine-rich protein that can bind a variety of heavy metal ions with its cysteine residues (Hamer 1986; Kagi and Schaffer 1988; Mercogliano and DeRosier 2007). To avoid the possible heavy metal toxicity or its influence on the behavior of target proteins, the cells were treated with the heavy metal during EM sample preparation (Morphew *et al.* 2015). However, the preservation of the cellular ultrastructure and the low tolerance of metallothionein to strong chemical fixation are still mutually exclusive. Another kind of clonable tag is based on the oxidization of 3,3′-diaminobenzidine (DAB). The contrast comes from the enriched osmium tetroxide that is recruited by the osmiophilic DAB polymer. According to the type of oxidation, these tags can be further classified into photo-oxidation tags, which include fluorescent protein (FP) (Grabenbauer *et al.* 2005; Meißlitzer-Ruppitsch *et al.* 2008), resorufin arsenical hairpin (ReAsH) (Gaietta *et al.* 2002), and mini singlet oxygen generator (miniSOG) (Shu *et al.* 2011), or they can be classified as enzyme-based oxidation tags, which include horseradish peroxidase (HRP) (Connolly *et al.* 1994; Li *et al.* 2010) and enhanced ascorbate peroxidase (APEX/APEX2) (Martell *et al.* 2012; Lam *et al.* 2015 *)*. Considering the low-yield efficiency of singlet oxygen by FPs as well as the relative low EM contrast, the usage of FPs is limited (Su *et al.* 2010). ReAsH works based on the reaction between tetracysteine tag and biarsenical compounds (Adams *et al.* 2002), which yields a problem of nonspecific labeling. Moreover, one general concern for the photo-oxidation-based tags is the inaccessibility of light in a deep tissue. Although the enzyme-based tags take advantage in dealing with thick tissue, the targeting capabilities of HRP in cytosol limit their usage (Hopkins *et al.* 2000). However, APEX/APEX2 has become a promising clonable EM tag because it can not only preserve cellular ultrastructure well but also give excellent EM contrast, regardless of the thickness of the specimen and the localization of target proteins (Martell *et al.* 2012). It is important to note that APEX2 is an A134P mutant of APEX with improved kinetics, thermal stability, heme binding, and resistance to high H_2_O_2_ concentrations (Lam *et al.* 2015).

Volume electron microscopy technique can study cellular ultrastructure in three dimensions (3D), which provides more information compared to the 2D projection or single slice. The transmission electron microscopy (TEM)-based volume EM methods include electron tomography (ET) (Hart 1968; Koster *et al.* 1992), serial sectioning followed by TEM (ssTEM) (Harris *et al.* 2006), and serial section ET (Ladinsky *et al.* 1999). The scanning electron microscopy (SEM)-based volume EM methods include serial section SEM (ssSEM) (Horstmann *et al.* 2012), serial block face scanning electron microscopy (SBF-SEM) (Denk and Horstmann 2004), and focus ion beam scanning electron microscopy (FIB-SEM) (Knott *et al.* 2008). For a small volume (50 × 50 × 30 μm^3^), FIB-SEM approach provides an efficient and automatic way to get the 3D ultrastructure of specimen with a higher axial resolution in comparison to serial section-based techniques and SBF-SEM (Peddie and Collinson 2014; Li *et al.* 2016).

As a highly dynamic organelle in eukaryotic cells, mitochondria perpetually divide, fuse, and move in response to ever-changing physiological demand of the cells (Youle and Van Der Bliek 2012). There are many proteins involved in mitochondrial dynamics and regulation. In mammalian cells, mitochondrial dynamics proteins 49 and 51 kDa (MiD49 and MiD51, respectively) (Palmer *et al.* 2011), fission protein 1 (Fis1, and hFis1 for human homologues) (Yoon *et al.* 2003), and mitochondrial fission factor (Mff) (Otera *et al.* 2010) are mitochondrial outer membrane proteins and play an important role as receptors of dynamic-related protein 1 (Drp1), which drives mitochondrial fission (Smirnova *et al.* 2001). Mitofusin 1 and 2 (Mfn1 and Mfn2, respectively) control the fusion of mitochondrial outer membrane (Santel and Fuller 2001) while OPA1 in both short and long forms play the central role of mitochondrial inner membrane fusion (Song *et al.* 2007). Overexpression of these proteins in mammalian cells will induce significant clustering of mitochondria, which was revealed from fluoresce microscopy (Griparic *et al.* 2004; Huang *et al.* 2007; Otera *et al.* 2010; Zhao *et al.* 2011; Liu *et al.* 2013). However, there was no information available on how these proteins are localized within the cluster and how these clustered mitochondria look like in 3D space.

In the present study, we developed a 3D localization EM method (APEX2–FIB-SEM) by combining FIB-SEM and APEX2 techniques to enable the correlation of protein localization information and cellular ultrastructure in the 3D space of a single cell with a high resolution. We utilized this approach to study the 3D localization of mitochondrial fusion- and fission-related proteins, including MiD51, MiD49, hFis1, Mff, and Mfn2.

## Results

### Workflow of APEX2–FIB-SEM

**Fig. 1:**
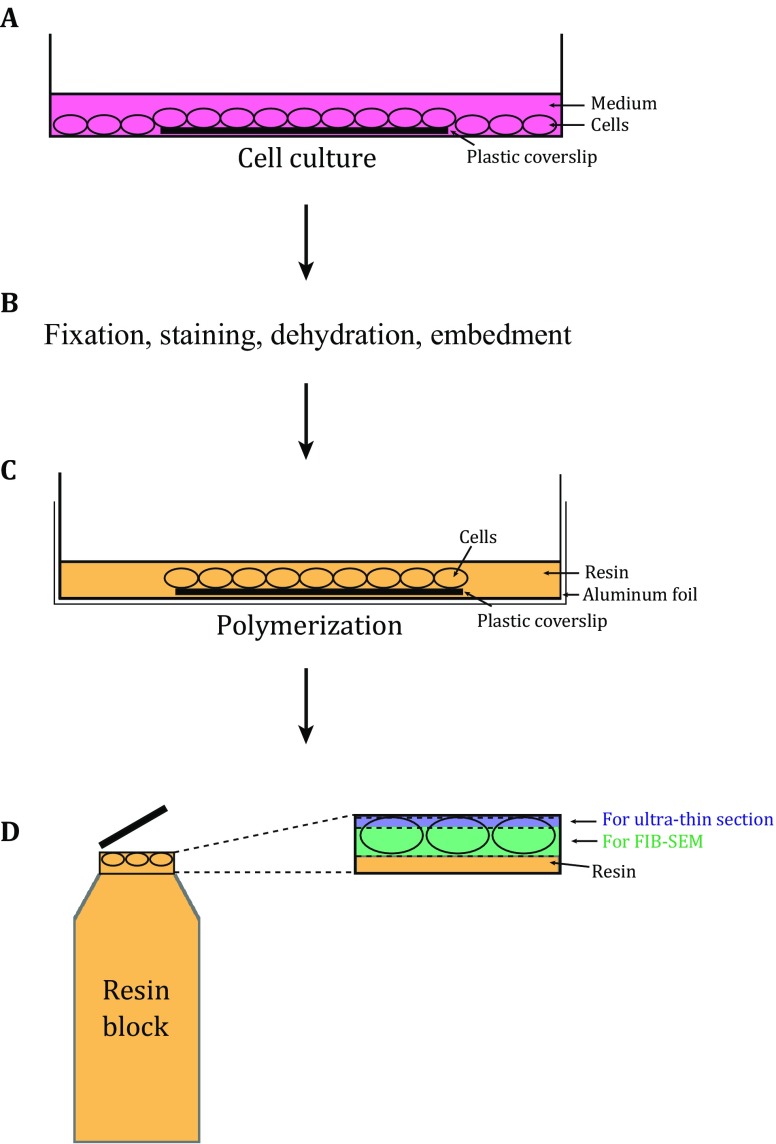
The scheme for APEX2–FIB-SEM. **A** The cells are cultured on sterile plastic coverslips in dishes, and transfected with the target genes fused with APEX2. The cells on the plastic coverslip are used for further EM sample preparation. **B** The cells on the plastic coverslip undergo fixation, DAB staining, dehydration, and resin embedment. **C** Before polymerization, the plastic coverslip with cells is transferred to the dish placed with aluminum foil. The cell side of the plastic coverslip is facing up. Fresh resin with accelerator is added to cover the cells. **D** After polymerization, the block is separated from aluminum foil and plastic coverslip, and the areas with staining patterns are cut off and stuck on the tip of an empty resin block. In order to reserve enough sample for FIB-SEM (colored with green), only three to five slices (less than 80 nm) are sectioned from the block (colored with blue) for TEM screening

The gene of the target protein with APEX2 fused at the N or C terminus was cloned into an appropriate vector (*e.g.*, pcDNA3 here) for transfection. The cells (*e.g.,* HEK 293T cells here) were cultured on a sterile plastic coverslip within a culture dish (Fig. 1A). After transfection and further culturing, the cells were fixed, stained with DAB, dehydrated, and then embedded in resin as previously reported (Fig. 1B) (Martell *et al.* 2012). Considering the reproducibility, we recommend to use the commercial DAB Kit (*e.g.*, CWBIO here) for DAB staining. Various embedding molds can be used for the polymerization of the resin-embedded cells with coverslip as long as they can be separated easily without damaging the cells (Fig. 1C). After polymerization, the plastic coverslip was removed carefully and a few ultrathin sections from the surface of the resin block were checked by TEM to screen for the optimally stained sample with well-preserved ultrastructure that will be further imaged by FIB-SEM. Only three to five slices were sectioned from the surface of the resin block and the thickness of each section was less than 80 nm to make sure there were still enough samples for subsequent FIB-SEM experiments (Fig. 1D). The details of the workflow are described in “Materials and methods”.

### 3D Localization of Mitochondrial Dynamics-Related Proteins

**Fig. 2:**
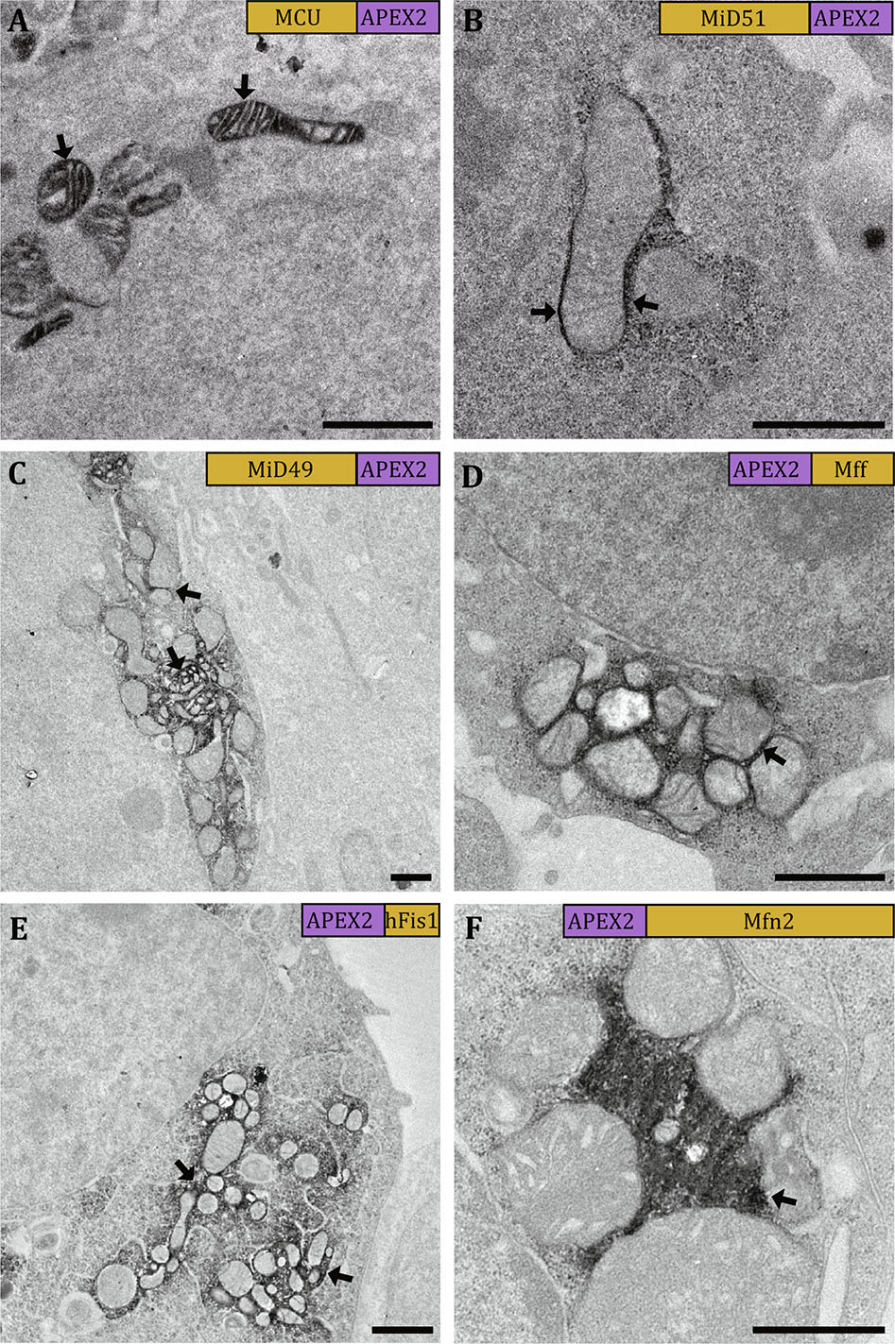
Visualizing the staining patterns in 2D by TEM. The micrographs show cells expressing MCU-APEX (**A**), MiD51-APEX2 (**B**), MiD49-APEX2 (**C**), APEX2-Mff (**D**), APEX2-hFis1 (**E**), and APEX2-Mfn2 (**F**). The staining areas with strong EM contrast indicate the locations of the target proteins (black arrows). The labels in the top right corner of each micrograph show the relevant position between the target protein and the tag as well as their relevant sizes. Scale bars, 1 μm

We first utilized mitochondrial calcium uniporter (MCU) as a positive control that was used in the previous APEX study (Martell *et al.* 2012) to validate the performance of APEX/APEX2 staining in our system. In our TEM experiment, MCU-APEX gave a strong EM contrast within mitochondrial matrix (Fig. 2A), which was consistent with the previous report (Martell *et al.* 2012) and showed that APEX worked well in our workflow.

In our further experiments, we selected APEX2 instead of APEX since APEX2 was more active due to its better resistance to high H_2_O_2_ concentration *(*Lam *et al.* 2015). By examining ultrathin sections in TEM, we found that the constructs MiD51-APEX2 (Fig. 2B), MiD49-APEX2 (Fig. 2C), APEX2-Mff (Fig. 2D), APEX2-hFis1 (Fig. 2E), and APEX2-Mfn2 (Fig. 2F) could yield a good EM contrast at the reasonable positions. These constructs were then selected for the subsequent FIB-SEM experiments. It is to be noted that, for the constructs Mff-APEX2, hFis1-APEX2, and Mfn2-APEX2, we could not find reasonable staining patterns in TEM micrographs. We speculated that it is due to the fusion of APEX2 in the proximity of the transmembrane helices of the target proteins, which induced the failure of their proper localizations in mitochondrial outer membrane (see also in “Discussions”).

**Fig. 3:**
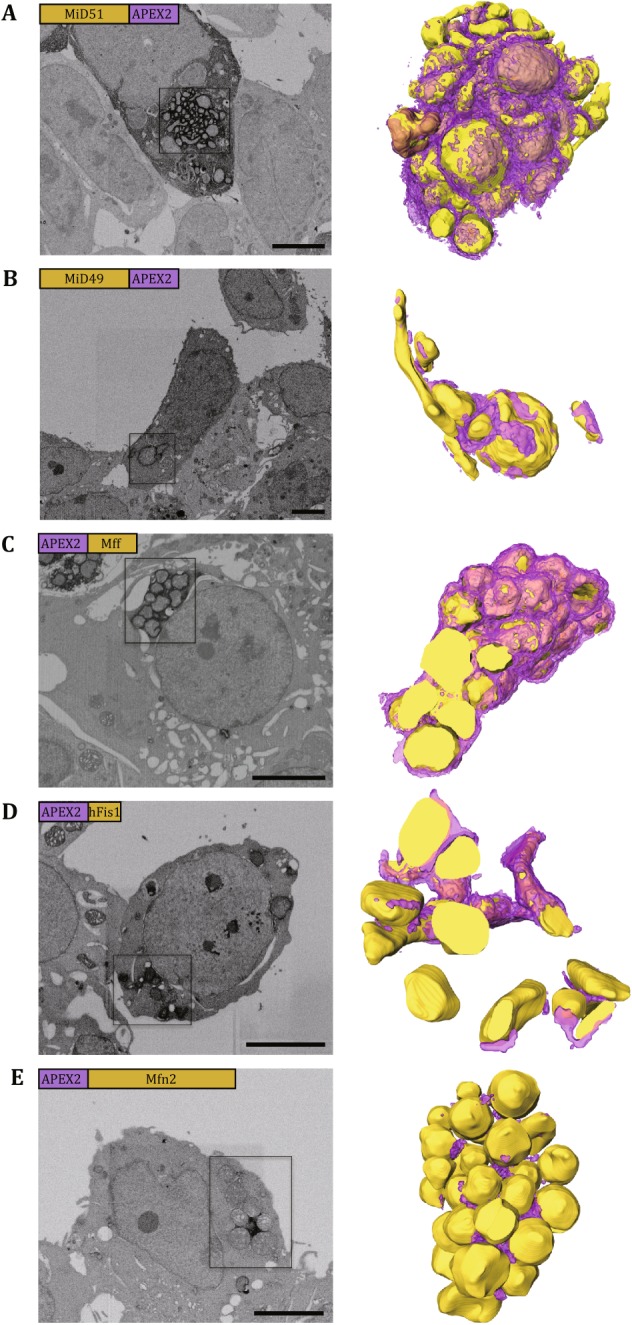
Visualizing the staining pattern in 3D by FIB-SEM. The figure panels show cells expressing MiD51-APEX2 (**A**), MiD49-APEX2 (**B**), APEX2-Mff (**C**), APEX2-Fis1 (**D**), and APEX2-Mfn2 (**E**). The left columns display the representative SEM micrographs from each volume of EM data, and the right columns display the 3D rendering of boxed areas of the left with the yellow spheres for mitochondria and purple layers for APEX2-induced EM contrast. The labels in the top left corner of each micrograph show the relevant position between the target protein and the tag as well as their relevant sizes. Scale bars, 5 μm

MiD51/49 has been found to recruit Drp1 into mitochondria, and overexpression of MiD51 or MiD49 induced mitochondrial elongation, which was thought to be the phenotype of mitochondrial fusion (Zhao *et al.* 2011; Liu *et al.* 2013). We also consistently found that mitochondria appeared as elongated tubulars or compact clusters in the cells overexpressing MiD51/49-APEX2 (Figs. 2B, C and 3A, B). However, the mitochondrial fusion phenotype induced by overexpression of MiD51/49 varied from cells to cells by the comparison of the EM images of cells expressing MiD51-APEX2 (see Figs. 2B and 3A) or those expressing MiD49-APEX2 (see Figs. 2C and 3B). We therefore speculated that the mitochondrial fusion phenotype induced by MiD51/MiD49 is highly dependent on their expression level (see also in “Discussions”). Through investigation of the 3D volume of the clustered mitochondria (Fig. 3A and B; see also Movies S1 and S2), we clearly found that the target proteins MiD51/MiD49 were largely localized at the interface among mitochondria with reduced amount of proteins surrounding the peripheral of the mitochondrial cluster. This observation implied how MiD51/MiD49 induced mitochondrial clustering by recruiting Drp1.

Mff was thought to be an essential factor for mitochondrial recruitment of Drp1 during mitochondria fission (Otera *et al.* 2010). In the cells with exogenous expression of APEX2-Mff, the mitochondria appeared in circular shapes and formed compact clusters (Figs. 2D and 3C) in consistency with the previous finding (Otera *et al.* 2010). Through investigation of the 3D volume of the clustered mitochondria, we clearly found that Mff was not only rich in the areas of mitochondrial connection, but also presented at the periphery of the mitochondrial cluster (Fig. 3C; see also Movie S3).

Fis1 was the first proposed Drp1 receptor and could induce the mitochondrial fission (Mozdy *et al.* 2000, James *et al.* 2003, Yoon *et al.* 2003). In the APEX2-hFis1-transfected cells, the clusters of vesicular mitochondria were found (Figs. 2E and 3D) in consistency with the previous study (Otera *et al.* 2010). The clustering phenotype also possibly varied in an expression level-dependent manner from cells (Fig. 2E) to cells (Fig. 3D). Through investigation of the 3D volume of the clustered mitochondria, we also found that hFis1 was located in high density at the interface of clustered mitochondria with a significant amount at the periphery of mitochondrial cluster (Fig. 3D; see also Movie S4).

Mfn2 can mediate tethering and fusion of mitochondrial outer membrane during the mitochondrial fusion process (Koshiba *et al.* 2004; Meeusen *et al.* 2004), with both of its N-terminal and C-terminal domains exposed towards the cytosol (Rojo *et al.* 2002). In the cells overexpressing APEX2-Mfn2, the mitochondria formed clusters and most of mitochondria were in circular shapes (Figs. 2F and 3E), which was consistent with the previous description of grape-like aggregation (Huang *et al.* 2007). However, to our surprise, unlike the clusters induced by the four proteins above, here, the mitochondrial cluster induced by overexpressing APEX2-Mfn2 was relatively loose, especially at the inside region that was filled with strong staining signals. In addition, through investigation into the 3D volume of the clustered mitochondria, we found that Mfn2 was only located in the contact sites of clustered mitochondria, and no staining signal was found at the periphery of mitochondrial cluster (Fig. 3E; see also Movie S5). This finding was significantly different from those associated with mitochondrial fission-related proteins MiD51, MiD49, Mff, and hFis1.

## Discussion

In this study, we developed the APEX2–FIB-SEM approach to determine the target protein localization within 3D cellular ultrastructural context by combining two state-of-the-art techniques, APEX2 and FIB-SEM. By using APEX2–FIB-SEM, we successfully mapped the position of mitochondrial dynamics-related proteins (MiD51/49, Mff, hFis1, and Mfn2) when they were overexpressed. The clusters with tubular (MiD51/49) or circular (Mff, hFis1, and Mfn2) mitochondria were found in those cells. Through investigation of the staining patterns in the 3D volume data, we found Mff and hFis1 distributed widely around the surface of mitochondrial outer membrane, and they were consistent with MiD51/49 except that the staining signals of MiD51/49 were reduced at the periphery of mitochondrial cluster. However, the relatively loose mitochondrial clusters and extremely restrained staining patterns at the mitochondrial contact site suggested that the mechanism of the fusion phenotype induced by overexpressing Mfn2 was distinctive from that of fission factors.

For alpha-helically anchored mitochondrial outer membrane proteins, the targeting signal is confined to transmembrane domain and positive residues in its flanking regions (McBride *et al.* 1992; Waizenegger *et al.* 2003; Bruggisser *et al.* 2017). The failure of the constructs, such as Mff-APEX2, hFis1-APEX2, and Mfn2-APEX2, is probably due to the fusion of APEX2 to the terminal which is close to the transmembrane region, and perturbs their translocation.

Like tagged with APEX2, similar morphologies are present in cells overexpressing the above five proteins without APEX2 (Huang *et al.* 2007; Otera *et al.* 2010; Zhao *et al.* 2011; Liu *et al.* 2013), which indicates that these mitochondrial phenotypes are not tag-associated artifacts. Their ubiquitous distributions at the interface regions of clustered mitochondria suggest that these proteins participate in tethering the mitochondria together at the outer membrane surface.

The different expression levels of the exogenous genes may induce distinct phenotype of cells and different behavior of proteins. As a result, the comparison of cellular phenotypes and location of proteins should be made with approximate expression levels. With too high expression level, the EM contrast would appear at unreasonable areas, for example in Fig. 3A, the MiD51 directed staining is also found at cytosol. The dispersing signals may result from the accumulated mis-located fusion proteins in cytosol.

In addition, the endogenous oxidases can also polymerize DAB and give false-positive signals, such as the cytochrome oxidase that is located in the intermembrane space of mitochondria (Seligman *et al.* 1968). However, most of the endogenous oxidases should have lower tolerance to strong chemical fixative than APEX/APEX2, which is the prerequisite to ensure APEX/APEX2 can work without interruption from endogenous oxidases. As a result, the fixation level should be well regulated to ensure that the endogenous oxidases are fully inactive without disrupting the activity of APEX/APEX2. In our experiment, the detached and pelleted cells were also tested for DAB staining and EM sample preparation, and uninterpretable staining was found at the mitochondrial intermembrane space and endosome matrix (data not shown). Moreover, staining was also found in the cells without transfection. However, the false-positive staining was missing with the same procedures for the monolayer cells. So the protocol of fixation and staining in this study is suitable for monolayer cells, and slight modification should be done in the fixation step for other kinds of samples.

## Materials and Methods

### Plasmids

All the genes were cloned into the pcDNA3.0 vector with five Myc tags near 3′ end of the multiple cloning site, respectively. All of them were inserted before c-Myc tag with a terminator. The A134P point mutation of APEX which turned APEX to APEX2 was created using extension overlap PCR. MiD51/49 and MCU were fused at the N-terminal of the APEX/APEX2, and Mff, hFis1, and Mfn2 were fused at both N- and C-terminals of the APEX2. MiD51-APEX2, MiD49-APEX2, hFis1-APEX2, Mfn2-APEX2, and MCU-APEX were cloned between BamH I and EcoR I sites. Mff-APEX2, APEX2-Mff, APEX2-hFis1, and APEX2-Mfn2 were cloned into Kpn I and BamH I sites. These fused genes were generated by overlap extension PCR, and the linker sequence between them was 5′-CTGGACAGCACC-3′. The fused genes were inserted into vector using standard restriction cloning methods and verified by nucleotide sequencing.

### Cell Culture and Transfection

HEK 293T cells were cultured in Dulbecco’s modified eagle medium (DMEM, Corning) supplemented with 10% (*v*/*v*) fetal bovine serum (Gibco). The cultures were kept in the condition of 5% CO_2_ and 37 °C. After culturing, the cells were placed into a 12-well plate (Corning) with each well having 200,000 cells approximately. It is to be noted that, before plating cells, a resized sterile plastic coverslip (Thermanox) was placed on the bottom of each well. After additional 24 h culturing, the cells were transfected by X-treme HP (Roche technologies), and then fixed after another 48–72 h.

### Sample Preparation for Electron Microscopy

The DAB staining and EM sample preparation were as previously reported (Martell *et al.* 2012) with slight modification. The plastic coverslip with transfected cells were fixed by 2.5% glutaraldehyde (Electron Microscopy Science) for 1 h on ice. Then they were washed by phosphate-buffered solution (PBS, pH 7.4) three times. Then the cells were incubated at 20 mmol/L glycine in PBS for 5 min, and rinsed by chilled PBS 3 × 5 min. DAB Kit (CWBIO) was used to stain cells for 15 min, followed by 3 × 2 min rinses in chilled PBS. The formation of DAB polymer could be checked by transmission light microscope. The cells were further post-fixed and stained by 2% osmium tetroxide (Electron Microscopy Science) for 30 min, and rinsed by distilled water 3 × 5 min. Then 2% aqueous uranyl acetate (Electron Microscopy Sciences) was placed on cells overnight. Thereafter, cells were gradient-dehydrated in ethanol series (30%, 50%, 70%, 80%, 95%, 100%) and 100% acetone. During the dehydration process of using 100% acetone, cells were transferred from ice temperature to room temperature. Cells were embedded by diluted Epon 812 (Electron Microscopy Sciences) in acetone (25%, 50%, 75%) for 2 h each and then by 100% resin three times with each time for 2 h. Before the resin polymerization step, the plastic coverslip with cells was transferred to a six-well plate coated with aluminum foil: the aluminum foil on the bottom of plate needs to be as flat as possible. The surface of plastic coverslip with cells was upward, and fresh resin with accelerator was added into the plates to cover the cells. The polymerization was performed at 60 °C for 48 h. Then the blocks could be taken out from the six-well plate and carefully separated from the aluminum foil and plastic coverslip. The cells should be near the surface of the blocks. Before the following steps, the block could be observed under a light microscope and the areas with significant staining signals should be cut off and stuck on the tip of a blank resin block that was polymerized in standard flat embedding mold (Electron Microscopy Sciences).

### Transmission Electron Microscopy

The blocks were trimmed and sectioned into 80 nm using an ultramicrotome (Leica EM UC6). Electron micrographs were recorded using FEI Tecnai spirit TEM (Thermo Fisher Scientific) operated at 120 kV.

### FIB-SEM and Image Processing

The block with the target regions was glued onto a 45° inclined aluminum sample stub with conductive silver paint. To avoid the charging artifacts, the blocks were coated with a thin-layer of carbon. The data were collected by FEI Helios NanoLab 600i Dual beam SEM (Thermo Fisher Scientific). The platinum layer was deposited on the region of interest with a gas injection system to protect the specimen surface from ion beam-induced damage. The accelerating voltage of FIB was 30 kV, and the beam current was set to 0.79 nA with the milling thickness of 15 or 20 nm for different volume data. The series micrographs were recorded using an accelerating voltage of 2 kV, a current of 0.69 nA, and a dwell time of 8 μs. The FIB-SEM data were aligned, reconstructed, and segmented by the Amira software (Thermo Fisher Scientific).

## Electronic supplementary material

Below is the link to the electronic supplementary material.

Supplementary material 1 (PDF 6 kb)

Supplementary material 2 (MPG 12424 kb)

Supplementary material 3 (MPG 9802 kb)

Supplementary material 4 (MPG 18334 kb)

Supplementary material 5 (MPG 18226 kb)

Supplementary material 6 (MPG 10834 kb)

## Abbreviations

3D: Three dimensions
APEX: Enhanced ascorbate peroxidase
CMV: Cytomegalovirus
DAB: 3,3′- diaminobenzidine
Drp1: Dynamic-related protein 1
EM: Electron microscopy
ET: Electron tomography
FIB-SEM: Focus ion beam scanning electron microscopy
Fis1: Fission protein 1
FP: Fluorescent protein
HRP: Horseradish peroxidase
MCU: Mitochondrial calcium uniporter
Mff: Mitochondrial fission factor
Mfn: Mitofusin
MiD: Mitochondrial dynamics protein
miniSOG: Mini singlet oxygen generator
ReAsH: Resorufin arsenical hairpin
ssTEM: Serial section transmission electron microscopy
ssSEM: Serial section scanning electron microscopy
SBF-SEM: Serial block face scanning electron microscopy
TEM: Transmission electron microscopy
