# Supplementary material for: Determining the target protein localization in 3D using the combination of FIB-SEM and APEX2
Source: Biophys Rep. 2017 Nov 4;3(4):92–9. doi: 10.1007/s41048-017-0043-x (PMC5719812; doi:10.1007/s41048-017-0043-x)
Supplement: Supplementary file 1 — Supplementary material 1 (PDF 6 kb) [file 41048_2017_43_MOESM1_ESM.pdf]

Supplementary Movies Legends

Movie S1 A video of Z-slicing view of FIB-SEM reconstruction of cells transfected with MiD51-APEX2. The axial thickness is 15 nm and there are 260 slices in total.

Movie S2 A video of Z-slicing view of FIB-SEM reconstruction of cells transfected with MiD49-APEX2. The axial thickness is 20 nm and there are 130 slices in total.

Movie S3 A video of Z-slicing view of FIB-SEM reconstruction of cells transfected with APEX2-Mff. The axial thickness is 15 nm and there are 248 slices in total.

Movie S4 A video of Z-slicing view of FIB-SEM reconstruction of cells transfected with APEX2-Fis1. The axial thickness is 20 nm and there are 133 slices in total.

Movie S5 A video of Z-slicing view of FIB-SEM reconstruction of cells transfected with APEX2-Mfn2. The axial thickness is 20 nm and there are 198 slices in total.

Supplemental movies are available at
http://feilab.ibp.ac.cn/data/APEX2FIBSEM/.
